# The use of health-related quality of life (HRQOL) in children and adolescents as an outcome criterion to evaluate family oriented support for young carers in Germany: an integrative review of the literature

**DOI:** 10.1186/1471-2458-8-414

**Published:** 2008-12-17

**Authors:** Jörg große Schlarmann, Sabine Metzing-Blau, Wilfried Schnepp

**Affiliations:** 1 Institute of Nursing Science, Witten/Herdecke University, Stockumer StraSSe 12, 58453 Witten, Germany

## Abstract

**Background:**

Young people below the age of 18, whose lives are affected by looking after a relative with a disability or long-term illness, are called young carers. Evidence based family oriented support for young carers and their families in Germany is currently being developed. To allow for scientific evaluation, an outcome criterion needs to be chosen. Until today, there are no assessment instruments available, which focus on young carer's specific demands and needs. As HRQOL seems to be an adequate alternative outcome criterion, an integrative review of the literature was carried out to verify this assumption.

**Methods:**

The aim of the integrative review was to get information about a) the concept and the common definition of HRQOL in children, b) preferable HRQOL assessment techniques in children, and c) the relevance of HRQOL measures for the population of young carers. An additional aim of the review was to give advice on which instrument fits best to assess young carer's HRQOL in Germany. Searches were conducted in PubMed in order to obtain papers reporting about a) the development or psychometric assessment of instruments measuring HRQOL in children and adolescents up to the age of 18, and b) on the conceptual framework of HRQOL in children.

**Results:**

HRQOL is a multidimensional construct covering physical, emotional, mental, social, and behavioural components of well-being and functioning as subjective perceived by a person depending on the cultural context and value system one is living in. Young carer's problems and needs are well covered by these common domains of HRQOL. Since no specific HRQOL-measures are available to address young carers, a generic one has to be chosen which a) has been created for use in children, b) allows self- and proxy-report, and c) has good psychometric testing results. Comparing four generic measures with currently best published psychometric testing results, items of the KIDSCREEN cover young carer's specific problems most accurate.

**Conclusion:**

The KIDSCREEN questionnaires seems adequate to evaluate the intervention as their items cover young carer's needs and problems most accurate.

## Background

It is well described that chronic illness not only affects the person concerned, but also the entire familial system [1-6]. Psychological distress and physical demands of caregiving can seriously compromise the quality of life (QOL) of a family caregiver [7-9]. As children might be involved in caring for their relatives -or even become the primary care giver – their burden has to be taken into account as well. These young people below the age of 18, whose lives are affected by looking after a relative with a disability or long-term illness, are called young carers [10,11]. A UK census [12] identified the prevalence of young carers as 1.5 percent of all children below the age of 18, which means that approximately 175,000 children in the UK are affected [13]. There is no current evidence about the prevalence in other countries. Results of national surveys in the UK state that children are in average between 8 and 10 years old when they get involved in caring for a relative, while the avarage age of young carers is 12 years [14,15]. While 84% of the young carers spend less than 20 hours a week with caring tasks, 9% are looking after a relative up to 50 hours a week [12]. The kind of help comprises all areas of caring and housekeeping [11,15-20], and the amount of help ranges from assistance to sole responsibility [16,18,21,22]. According to Dearden and Becker [15], "housekeeping" and "general care" are the main activities of young carers to help their family members (table 1). Researchers from the UK refer to the vulnerability of families concerned and they predict that children will be affected in their whole development if the families stay without support [18,23]. Currently, there are more than 300 young carers projects available in the UK, where these children are supported and counseled [24].

**Table 1:** Type of activities according to Dearden and Becker [14]

	**below 5 years**	**5–10 years**	**11–15 years**	**above 15 years**
**housekeeping**	25% (n = 5)	55% (n = 401)	73% (n = 894)	81% (n = 264)

**general care**	10% (n = 2)	45% (n = 327)	56% (n = 691)	68% (n = 222)

**intimate care**	5% (n = 1)	11% (n = 79)	21% (n = 258)	34% (n = 111)

**emotional care**	20% (n = 4)	35% (n = 256)	44% (n = 540)	44% (n = 142)

**siblings care**	10% (n = 2)	6% (n = 41)	7% (n = 83)	6% (n = 18)

### Young Carers in Germany

In Germany, hardly anything is known about the situation of children, who are involved in caring for their relatives, and, as a consequence, there are no specific support services available. There is no current evidence about the prevalence in Germany, but if the British prevalence data [12] was adopted then there would be approximately 225,000 young carers in Germany. Young carer's personal and familial situation in Germany, their family's needs and expectations have recently been explored in a Grounded Theory study [25,26]. The aim of the study was to gain insight into experience and construction of familial care, in which children take over an active role, in order to work out a basis for the conception of specific family oriented support. One main focus of the study lies on effects on the children and their expectations in outside support. The study's results confirm that young carers might suffer from their situation in several ways [25,26]:

• having no one to talk to

• living in secrecy

• lack of freetime

• social isolation and loss of childhood

• problems in school and missing time in school

• strong parental attachment

• feelings of loneliness, sadness, fear and shame

• physical and mental exhaustion

These findings point out the need for support for families concerned. Therefore, the aim of a current study is to develop, implement and evaluate evidence based family oriented support for young carers and their families in Germany. The intervention's concept a) is based on Metzing's [25,26] results, b) is based on expert interviews with project leaders and young carers themselves, c) focuses on the individual needs of families and d) has to allow for scientific evaluation. The aim of family-oriented support for young carers and their families is to disburden their situation and reduce the risk of negative impact. This study is a project of the Nursing Research Network North-Rhine-Westphalia [27], and it is funded by the German Federal Ministry of Education and Research (BMBF, project funding reference number 01GT0619).

Although young carers projects are well established in the UK, hardly any project has been evaluated with a standardized assessment instrument.

## Aim

At present, there are no specific assessment instruments available, which focus on young carer's situation, demands and needs. In order to allow scientific evaluation, an outcome criterion measuring the effectiveness of specific support has to be chosen. As health-related quality of life (HRQOL) in children has become an important outcome indicator in evaluating health-care interventions, it may be well used for the evaluation of young carers support. To verify the assumption, an integrative review of the literature has been carried out. If the review confirms HRQOL to be suitable, an additional aim of the review is to give advice on which instrument fits best to assess young carer's HRQOL in Germany. In what follows, results of this review will be presented.

## Methods

The review follows the method described by Polit and Hungler [28]. The following questions were addressed to the literature:

• What is the concept and the common definition of HRQOL in children?

• How can HRQOL be assessed in children and which assessment-technique is preferable?

• Are the dimensions and items of existing HRQOL measures of relevance for young carers?

**Figure 1:**
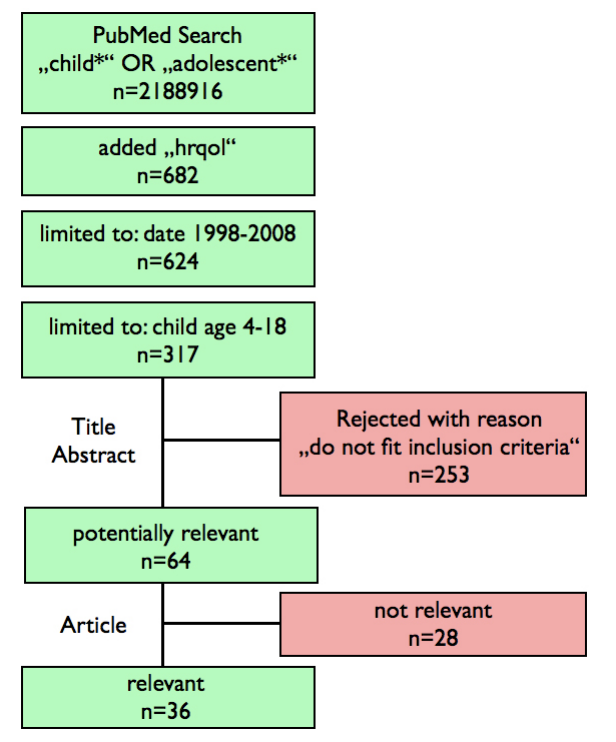
**Search strategy**. Flow chart of the search strategy conducted in PubMed.

A PubMed database search was carried out using keywords such as "children", "adolescent", in combination with "health-related quality of life", "quality of life", "HRQOL" and "QOL" (figure 1). In addition, references from eligible articles were hand-searched.

### Inclusion and Exclusion criteria

The search was restricted to documents written in English or German.

Articles published between 1998 and 2008 were included, if they reported about the development or psychometric assessment of instruments measuring HRQOL in children and adolescents up to the age of 18. Papers, reporting on the conceptual framework of HRQOL in children were also included. Documents were excluded if the measures used were not originally designed for use in children or adolescents.

#### Procedure

Documents identified by the search were checked for relevance by one reviewer (JgS). Data from documents considered eligible for inclusion was extracted using the software MAXqda. Content of documents was structured using the following categories: a) definition and concept of HRQOL, dimensions and factors of HRQOL, b) measurement instruments, development, country of origin, population, type of respondent, age group.

## Results

In sum, 317 papers were found, of which 64 fit the inclusion criteria. Of these, 36 were regarded as being relevant for this review. Five systematic reviews were found. Three focused on identification and evaluation of all available measures of HRQOL in children [29-31], two on conceptual framework of HRQOL in children [32,33]. These reviews gave valuable information about children's HRQOL in general, and were helpful for finding further important literature. In total, 43 documents were included in this review.

### Health-related quality of life in children and adolescents

Quality of Life (QOL) is a complex, abstract, and multidimensional concept which is difficult to define and has relevance to virtually all areas of human function [34]. HRQOL is a main part of QOL and is considered to be an important construct in describing one's overall condition within the health context [35-38]. Generally it is conceptualized as a multidimensional construct built up by several domains [39-42]. There is some consensus considering physical, emotional and social aspects of health to be core domains of HRQOL [43-45], which follows the WHO definition of health as a state of complete physical, mental, and social well-being, and not merely the absence of disease or infirmity [46].

Nevertheless, the overall quantity of domains differs in the literature. For example, "behavioural, cultural, and psychological dimensions" as well as "a global perception of health and well-being" are regarded as important domains of HRQOL [30,36]. Depending on the population under study, HRQOL domains consist of several various specific dimensions or factors [47]. For example, physical factors might include aspects such as selfcare, pain, or mobility, while social factors might include aspects such as friends, work, or family. These differences in quantity of domains and dimensions lead to widely varying definitions of HRQOL. Based on a commonly accepted definition by the WHO Quality of Life group [48], the following operational definition as stated by von Rüden [35] is used in this paper:

HRQOL is a multidimensional construct covering physical, emotional, mental, social, and behavioural components of well-being and functioning as subjective perceived by a person depending on the cultural context and value system one is living in.

Since this definition applies to HRQOL of any person, the specific aspects of a child's life lead to different extracts and weightening of domains and factors compared to adolescents or adults [41]. Regarding "social components" for example, children state "family", "peer group" and "school" to be important factors [41,49,50]. While younger children consider "family" as the most important, adolescents highlight "peers" [49,50]. Furthermore, in comparison with adults, children have only limited capabilities to move from disadvantageous environments [36]. Thus, social context might have more influence on children's HRQOL than on adult's.

#### HRQOL research in children

According to Ravens-Sieberer et al. [40,51] the development of HRQOL research in children occurred in three phases. The first phase in the late 1980s was concerned with the theoretical concept of HRQOL in children, especially in contrast to adults. During the second (still ongoing) phase, which started in the early 90s, several HRQOL measures for children have been developed. The third phase, from 1995 onwards, emphasizes the application of these measures in clinical and epidemiological studies (ibid.). In the meantime, HRQOL in children has become an important outcome indicator a) in evaluating health-care interventions, b) in identifying health inequalities, c) in detecting subgroups at risk within the general population, and is d) used in epidemiological studies and health surveys [30,51]. Knowledge about children's HRQOL is of specific interest in public health research as it is the basis for HRQOL in adulthood [43]. Therefore, research assessing the HRQOL status of children has been carried out in many countries. Findings show that there are age-, gender- and socioeconomic-related differences in children's HRQOL. Children up to the age of 12 report higher HRQOL than adolescents [40,43,52]. As they get older, adolescent girls show lower HRQOL than boys, especially in the domains of emotional and physical well-being (ibid.). In general, children and adolescents with a high familial socioeconomic status report higher HRQOL than those less affluent [40,53].

### Measuring HRQOL in children and adolescents

At the beginning of HRQOL research in children, measures for adults had been modified to fit for children [51,54]. Today, there is a clear agreement that instruments originally developed for adults are not applicable to assess children's HRQOL [55]. Besides a different understanding of health and health-related domains and dimensions, children's emotional and cognitive development have to be taken into account [40,56]. Therefore, measures especially for the use with children have been created.

#### Development of measures

HRQOL can be considered as a latent theoretical construct which cannot be measured directly but only indirectly using indicators [35,57]. While most of the early instruments were based on expert opinions about important HRQOL domains [50], several new questionnaires preferred the use of focus groups with children to reflect their opinions and ideals of HRQOL in order to identify relevant domains and dimensions [29,36,50,58]. However, due to children's cognitive development and rising awareness, it has to be taken into account that their concept of health changes as they mature [59]. Addressing these developmental differences, measures are created in multiple forms, each designed for a different age group [41], since item statements have to consider the cognitive developmental level of the children at different ages [41,42,51]. This means that tools such as likert scales need to be considered from the perspective of the child's ability to understand and may require adaption using pictograms or smileys [51]. In addition the number of questions should also be limited as younger children can process up to less items than older [41,42]. Another possible approach is to identify relevant items which are understood by and function in comparable ways across different age groups [36].

As the process of HRQOLresearch in children went on, it has been regarded as a limitiation that measures were developed in only one country and then were translated for use in other countries without regard to cultural differences [60,61]. As the connotation of HRQOL shows cultural differences (ibid.), these instruments cannot be used offhand for comparing populations across different countries without difficulties. A translated version must therefore undergo new tests for validity and reliability before it can be relied upon for usage in that country or culture. To avoid this problem, it has been recommended to simultaneously develop a measure across different countries using focus groups [40,50,62].

#### Generic and specific approach

There are two general types of HRQOL instruments: generic and specific ones. Generic measures are used to get information about HRQOL on healthy as well as on ill children in different populations, conditions, and settings. Thus, these results can be compared across groups [29,30]. Specific measures are designed to be valid for a specific disease or population and aim to gather information on specific disease-symptoms or health-problems [63]. Compared to generic, specific measures tend to be more sensitive to changes arising from changes in conditions and may, therefore, be more effective in identifying intervention effects [41,63]. On the other hand they cannot be used to compare HRQOL across conditions and settings. Some measures are now being developed incorporating both a generic core and disease-specific modules [29]. A recent review [30] identified a total of 94 instruments that focus on children. Of these, 30 are generic and 64 are disease-specific (ibid.). Nevertheless, authors still complain about the limited availability of specific instruments for certain diseases [29,31]. At this time, there are no specific instruments available for measuring HRQOL of young carers. Thus, generic instruments are warranted for the use in this population.

#### The proxy-problem

There are two main approaches to assess HRQOL in children: self- and proxy-report. By using self-report, the child's self-perception of HRQOL is measured. While in the past, children often were regarded as unreliable respondents due to their cognitive immaturity, limited social experience, and continued dependency [64], early measures were based on data provided by parents or other proxies (e.g. medical staff) [44,55,65,66]. Thus, the proxy's *perception *of a child's HRQOL is assessed. These two approaches cannot be considered to measure identical constructs and research findings confirm a moderate correlation only [42,66-69]. The level of agreement between parents and children appears to depend on the observability of a certain dimension, with generally good agreement reflecting physical dimensions and poor agreement reflecting social and emotional dimensions [65,66,68]. Davis [42] states that proxy-discordance is due to differences in parent's and children's response styles, interpretation of items, and reasons for answering. Besides this, Cremeens et al. [68] concern that artefact of statistical methods may have also caused proxy-disagreement in former research.

Nevertheless, according to the concept of HRQOL, the individual's own subjective perception should be measured to get valid data. This is true for children as well as for adults. Recent research shows, that children as young as eight [50,70] and even at the age of six years [44,70,71] can reliably and validly self-report their HRQOL status *if *the questionnaire is age- and cognitive-appropriate. In detail, measures for young children should a) address their writing and reading skills [51], b) consider alternative assessment methods as pictograms or smileys [51], and c) avoid Likert-Scales in order to prevent extreme answers [41,42].

Because neither the child's self report nor the parent's proxy report is without bias, Eiser [66] suggests that obtaining information from both may provide the most complete picture of HRQOL.

#### Selecting an HRQOL instrument

When selecting a HRQOL instrument, it is important to consider whether the questionnaire suits the purpose of the intervention, whether it covers important domains and dimensions relevant to the context and whether it fits the age group under study [30,72]. In addition, there should be sufficient psychometric testing of the instrument.

### Relevance of HRQOL dimensions and measurement items for use in young carers

According to the definition as stated by von Rüden [35], young carer's problems and needs (as worked out by Metzing [25,26]) are well covered by the core domains of HRQOL (table 2). Their problems are mainly related to the social domain and its factors, followed by the emotional and mental domain. One problem is covered by the physical domain.

**Table 2:** Young carer's problems [25,26] covered by HRQOL domains/factors

**Young carer's problems**	**HRQOL domains**
having no one to talk to	social domain: peers, emotional domain

lack of freetime	social domain: leisure, emotional domain

living in secrecy	social domain: social life and support

social isolation	social domain: social life and support

decreasing school performance	social domain: school, mental domain

strong parental attachment	social domain: family/autonomy

loneliness, sadness, fear and shame	emotional domain: moods

physical and mental exhaustion	physical domain: functioning, mental domain

Solans et al. [30] present a detailed overview of available generic and specific measurements including information about covered HRQOL domains and psychometric testing. The PubMed search added no additional instrument to this list. According to Solans et al. [30], there are four generic measures with the best published psychometric testing results: CHQ [73], KINDL^*R*^[58], KIDSCREEN [36], and PedsQL 4.0 [74]. Each of these instruments covers nearly all domains and factors, which are relevant for young carers (table 3), and they also have a German version (the KINDL^*R *^is an original German measure). The CHQ only omits the factor "leisure", both KINDL^*R *^and KIDSCREEN omit the mental dimension, and PedsQL omits the mental dimension and the factor "family".

**Table 3:** HRQOL domains/factors covered by measures

**domain/factor**	**CHQ**	**KIDSCREEN**	**KINDL**^*R*^	**PedsQL**
peers	x	x	x	x

moods	x	x	x	x

leisure	-	x	x	x

social life	x	x	x	x

school	x	x	x	x

family/autonomy	x	x	x	-

mental	x	-	-	-

physical	x	x	x	x

Comparing the instruments' items in detail, as shown in additional file 1, there are differences in how accurate the specific problems of young carers are addressed. Items of the **CHQ **don't address the problems "lack of freetime" and "physical and mental exhaustion". The item "I feel lonely" allows to refer to the problems "having no one to talk to", "living in secrecy", "social isolation" and "loneliness, sadness, fear". The problem "living in secrecy" is only addressed indirectly by the items "I lied/cheated" and "I feel lonely".

The **KIDSCREEN **allows to link its items to all problems of young carers. Four items are related to more than one problem: The item "have you had enough time for yourself" may be an indicator for the problems "lack of freetime" and "parental attachment", while "have you spent time with your friends" points to "lack of freetime" and "social isolation". The item "have you felt so bad that you didn't want to do anything" refers to "loneliness, sadness, fear" and "physical and mental exhaustion", while the item "have you felt lonely" allows for the same multiple link as CHQ's "I feel lonely". The problem "living in secrecy" is only allusively addressed by the items "have you felt lonely" and "have you been able to rely on your friends".

Items of the **KINDL**^*R *^allow for covering all problems of young carers. Comparable to KIDSCREEN, two of KINDL^*R*^'s items refer to several problems. The item "I played with my friends" points to the problems "lack of freetime" and "social isolation", while the item "I felt alone" allows for the same multiple link as KIDSCREEN's "have you felt lonely" and CHQ's "I feel lonely". Comparable to CHQ and KIDSCREEN, the problem "living in secrecy" is only allusively covered by the single item "I felt alone".

The **PedsQL **does not represent the problems "having no one to talk to", "living in secrecy" and "parental attachment". Two of its items point to multiple problems. While the item "I cannot do things that other kids my age can do" refers to the problems "lack of freetime" and "social isolation", the item "I forget things" indicates decreased ability to perform at school and is an indirect measure of the effect of "physical and mental exhaustion".

To summarise, both CHQ and PedsQL don't match two problems of young carers, whereas items of KIDSCREEN and KINDL^*R *^allow for addressing all of them (additional file 1). Furthermore, KIDSCREEN seems to be more accurate to address young carer's problems than the other instruments. The problem "having no one to talk to" is indirectly covered by CHQ's item "I feel lonely" and KINDL^*R*^'s "I felt alone" while KIDSCREEN additionally asks "have you been able to talk to your parents when you wanted to" and "have you and your friends helped each other".

The problem "lack of freetime" is covered allusively by KINDL^*R*^'s questions "I was bored" and "I played with friends", while PedsQL's item "I cannot do things that other kids my age can do" allows for an indirect link. In contrast, KIDSCREEN directly asks "have you had enough time for yourself" (additional file 1).

Regarding the problem of strong "parental attachment", the CHQ only assess the family's general "ability to get along with one another". The KINDL^*R*^asks whether "my parents stopped me from doing certain things", which can be understood as active parental interdictions. KIDSCREEN's item "have you been able to do the things you want to do in your free time" however allows to cover leisure activities which are unfeasible due to parental impairment.

## Discussion

testing results. The literature supports the use of HRQOL with young carers as its domains cover the problems they experience. Thus, HRQOL seems to be a suitable outcome criterion measuring the effectiveness of special young carers support. As HRQOL in children has become an important outcome indicator in evaluating health-care interventions, there are several measurements available. Since no specific HRQOL-measures are available to address the specific situation of young carers, a generic one has to be chosen for use in this population. The literature advises to select a measure, which a) has adequate psychometric testing results, b) allows for self and proxy assessment, c) has been devloped in the country of origin or crosscultural, and d) which items cover important domnains relevant to the context.

According to Solans et al. [30], the CHQ [73], KIDSCREEN [36], KINDL^*R*^[58], and PedsQL 4.0 [74] are generic measures with the best published psychometric testing results. In addition, all of these fulfill the request of self and proxy assessment. Concerning their developmental process, there is a difference between the four. While CHQ, KINDL^*R *^and PedsQL were designed within a single country and have been translated into several languages afterwards, only KIDSCREEN was developed simultaneously across 13 European countries (Germany included). The most important difference between the four instruments was found while comparing how sensitive the instruments' items cover the context under study. As described before, KIDSCREEN turns out to be the most accurate instrument, and thus will be used for evaluating young carers support service. Nevertheless, some of young carer's problems are not directly matched by the instruments. For example, regarding the problem "having no one to talk to" it would be helpful to ask "do you have the feeling, that there is no one you can talk to". Concerning "living in secrecy", a question like "do you have to conceal something" would be adjuvant. This shows that although HRQOL measures allow for addressing young carer's problems, there is still a need to develop instruments which are desinged for use in this specific population.

This even rises the question, whether we need to broaden our understanding of outcome measures. In order to find an appropriate outcome criterion for family oriented support of young carers, we focused on individual HRQOL instruments. But if we adopt a family oriented perspective, an outcome criterion might need to address the family system as a whole. For example, for adult care givers, there are measures available which assess the impact and burden a chronically ill child has on the family (e.g. the "Impact on Family Scale" [75], which has a German version [76]). However, these measures are related to specific topics, and until today none of them focuses on the situation of young carers and their families. In addition, they are designed for the use in adults only. On the other hand, although young carers support needs to be family oriented, the focus of our current study still lies on the children, their experiences and well-being. Nevertheless, future research on instrument development for use in the population of young carers and their familie should consider a systematic approach.

## Conclusion

The literature approves HRQOL to be an adequate alternative criterion to evaluate the effectiveness of a support service for young carers. Comparing available HRQOL measures, the KIDSCREEN questionnaires fit best to assess young carer's HRQOL in Germany, as

• young carer's specific problems are well-covered by KIDSCREEN's items,

• it allows for both self- and proxy assessment,

• it shows good psychometric testing results,

• it was developed cross-culturally, including Germany.

Nevertheless, as some of young carer's specific problems are not directly addressed by current HRQOL measures, there is a need to develop instruments focusing on this specific population.

## Limitation

The manuscript is not a systematic but an integrative review. This is due to the reason that there are currently neither RCTs evaluating young carers support services nor assessment instruments focusing on young carers' specific situation available.

The literature search was carried out in PubMed only.

Only items of the four instruments with the best published psychometric testing results where analysed.

## Recommendation for future research

The results of the literature search show a lack of standardised measures designed for the use in young carers, and thus implicates three possible approaches: a) to develop independent instruments focusing on the specific problems of young carers, b) to develop specific young carers modules which can be connected to the generic core of available HRQOL-measures (e.g. the KINDL^*R*^), and c) to develop instruments for the use in children, which have a systematic view on the impact that chronic illness has on the entire family.

## Competing interests

The authors declare that they have no competing interests.

## Authors' contributions

JgS carried out the literature search, included and excluded documents, and wrote the manuscript. SMB and WS revised it critically for important intellectual content. All authors read and approved the final manuscript.

## Pre-publication history

The pre-publication history for this paper can be accessed here:



## Supplementary Material

Additional file 1 Young carer's problems covered by instrument items. Young carer's problems covered by items of CHQ, KIDSCREEN, KINDL and PedsQL Click here for file
