# Supplementary material for: The use of health-related quality of life (HRQOL) in children and adolescents as an outcome criterion to evaluate family oriented support for young carers in Germany: an integrative review of the literature
Source: BMC Public Health. 2008 Dec 17;8:414. doi: 10.1186/1471-2458-8-414 (PMC2625356; doi:10.1186/1471-2458-8-414)
Supplement: Additional file 1 — Young carer's problems covered by instrument items. Young carer's problems covered by items of CHQ, KIDSCREEN, KINDL and PedsQL [file 1471-2458-8-414-S1.doc]

Table 4: Young Carer's problems covered by instrument items

| **Young Carers prolems** | **CHQ-Items** | **KIDSCREEN-Items** | **KINDL^R^-Items** | **PedsQL-Items** |
| --- | --- | --- | --- | --- |
| **Having no one to talk to** | *-* „I feel lonely“[[1]](#footnote-2)1 | 2.6 „Have you felt lonely?“ ^1^  3.5 „have you been able to talk to your parents when you wanted to?“  4.3 „have you and your friends helped each other?“ | 2.3 „I felt alone“^1^ | *[no match]* |
| **Living in secrecy** | - „I lied/cheated“[[2]](#footnote-3)2  - „I feel lonely“^1,2^ | 2.6 „Have you felt lonely?“^1,2^  4.4 „have you been able to rely on your friends?“^2^ | 2.3 „I felt alone“^1,2^ | *[no match]* |
| **Lack of freetime** | *[no match]* | 3.1 „have you had enough time for yourself?“ ^1^  4.1 „have you spent time with your friends?“ ^1^ | 2.2 “I was bored” ^2^  5.1 „I played with my friends“^1^ | 3.4 „I cannot do things that other kids my age can do“^1^ |
| **Social isolation** | - „I had a hard time getting along with others“  - „I feel lonely“^1^ | 2.6 „Have you felt lonely?“ ^1^  4.1 „have you spent time with your friends?“ ^1^ | 2.3 „I felt alone“^1^  5.1 „I played with my friends“^1^  5.4 „I felt different from other children“ | 3.1 „I have trouble getting along with other kids“  3.2 „Other kids do not want to be my friend“  3.4 „I cannot do things that other kids my age can do“^1^ |
| **Decreasing school performance** | „I had a hard time paying attention“  „I did not do what my teacher or parent asked me to do“ | 5.2 „have you got on well at school?“  5.3 „have you been able to pay attention?“  5.4 „have you got along well with your teachers?“ | 6.1 „doing my schoolwork was easy“  6.4 „I worried about bad marks or grades“ | 4.1 „Its hard to pay attention in class“  4.2. „I forget things“^1^  4.3 „I have trouble keeping up with my schoolwork“  4.5 „I miss school to go to the doctor or hospital“ |
| **Parental attachment** | „In general, how would you rate your family's ability to get along with one another?“ | 3.1 „have you had enough time for yourself?“^1^  3.2 „have you been able to do the things you want to do in your free time?“  3.4 „have your parents treated you fairly?“ | 4.2 „I felt fine at home“  4.4 „my parents stopped me from doing certain things“ | *[no match]* |
| **Loneliness, sadness, fear, and shame** | „How much of the time do you: feel sad; feel like crying; feel afraid or scared; worry about things; feel lonely[[3]](#footnote-4)1; feel unhappy; feel nervous; feel bothered or upset; feel happy; feel cheerful“ | 2.1 „has your life been enjoyable?“  2.2 „have you been in a good mood?“  2.4 „have you felt sad?“  2.5 „have you felt so bad that you didn’t want to do anything?“ ^1^  2.6 „Have you felt lonely?“ ^1^ | 2.3 „I felt alone“^1^  2.4 „I felt scared“  3.3 „I felt pleased with myself“ | 2.1 „I feel afraid or scared“  2.2 „I feel sad or blue“  2.3 „I feel angry“  2.5. „I worry about what will happen to me“ |
| **Physical and mental**  **exhaustion** | *[no match]* | 1.2 „have you felt fit and well?“  1.5 „have you felt full of energy?“  2.5 „have you felt so bad that you didn’t want to do anything?“ ^1^ | 1.1 „I felt ill“  1.3 „I was tired and worn-out“  1.4 „I felt strong and full of energy“ | 1.8 „I have low energy“  4.2 „I forget things“1,[[4]](#footnote-5)2 |

1. ^1^ refers to more than one problem [↑](#footnote-ref-2)
2. ^2^ addresses problem allusively [↑](#footnote-ref-3)
3. ^1^ refers to more than one problem [↑](#footnote-ref-4)
4. ^2^ addresses problem allusively [↑](#footnote-ref-5)
